# Simultaneous Depression of Immunological Synapse and Endothelial Injury is Associated with Organ Dysfunction in Community-Acquired Pneumonia

**DOI:** 10.3390/jcm8091404

**Published:** 2019-09-06

**Authors:** Rosario Menéndez, Raúl Méndez, Raquel Almansa, Alicia Ortega, Ricardo Alonso, Marta Suescun, Ana Ferrando, Laura Feced, Jesús F. Bermejo-Martin

**Affiliations:** 1 Pneumology Department, Hospital Universitario y Politécnico La Fe/Instituto de Investigación Sanitaria (IIS) La Fe, University of Valencia, 46026 Valencia, Spain; 2 Centro de Investigación Biomédica En Red-Enfermedades Respiratorias (CibeRes, 58 CB06/06/0028), Instituto de salud Carlos III (ISCIII), Av. de Monforte de Lemos, 5, 28029 Madrid, Spain; 3 Hospital Clínico Universitario de Valladolid, Av. Ramón y Cajal, 3, 47003 Valladolid, Spain; 4 Group for Biomedical Research in Sepsis (BioSepsis), Hospital Universitario Río Hortega de Valladolid, Calle Dulzaina, 2, 47012 Valladolid, Spain; 5 Instituto de Investigación Biomédica de Salamanca (IBSAL), Paseo de San Vicente, 58-182, 37007 Salamanca, Spain; 6 Laboratory Department, Hospital Universitario y Politécnico La Fe, 46026 Valencia, Spain

**Keywords:** pneumonia, transcriptomic signature, endothelial

## Abstract

Rationale: A depressed expression of antigen presentation is, along with endothelial dysfunction, a recognized signature of severe community-acquired pneumonia (CAP). We aimed to evaluate the expression of a number of genes involved in the immunological synapse in non-critically ill CAP patients with or without organ dysfunction and to profile endothelial biomarkers such as proendothelin-1 (proET1) and proadrenomedullin (proADM). Methods: A nested study in a prospective cohort in CAP patients was performed. Expression levels of major histocompatibility complex class II DR alpha (HLA-DRA), CD40 ligand (CD40LG), CD3E, CD28, and inducible T-cell costimulator (ICOS) were quantified by using droplet digital polymerase chain reaction and endothelial biomarkers by immunofluorescence. Results: Ninety-four patients were included, 44.7% of whom had organ failure in one or more organs. A significant decrease in the expression of the five genes with increased levels of proadrenomedullin (proADM) and proendothelin-1 (proET1) was found in CAP with organ failure. The depressed expression of HLA-DRA (odds ratio (OR), 2.94), CD40LG (OR, 3.90), and CD28 (OR, 3.48) was independently associated with organ failure after adjustment for age, Charlson score, and severity. Conclusions. CAP with organ failure showed depressed expression of immunological synapse genes with increased levels of biomarkers denoting endothelial damage. Simultaneous profiling of immunological and endothelial signatures could help in the early identification of organ failure in CAP and in the implementation of personalized treatment.

## 1. Introduction

Community-acquired pneumonia (CAP) has an incidence of 3–5 cases per 1000 adults-year and, generally in the most severe episodes, causes morbidity and deaths worldwide, mostly in older patients and in those with comorbidities. Host adequate immune response requires microorganism identification for mounting an innate and adaptive response to eradicate microorganisms without damaging lungs and distal organs. In fact, sepsis appears when infection provokes life-threatening organ dysfunction caused by a dysregulated host response [1]. CAP is one of the main causes of sepsis, mainly due to Gram-positive microorganisms. Approximately one-third of admitted CAP patients have already developed organ dysfunction in one organ at diagnosis and 10% have organ dysfunction in two or more organs [2]. 

The mechanisms underlying the dysregulated response and organ dysfunction in CAP are not totally clear [3]. It has been reported that there are signatures denoting the presence of an inadequate immunologic response quite early in the infection process. Immunologic dysregulation and concomitant endothelial dysfunction are two features typically found in sepsis [4]. In fact, sepsis [5] and ventilator-associated pneumonia (VAP) are characterized by the depression of the immunological synapse [6]. However, there is scarce information of immunological signatures at the transcriptomic level in CAP in non-critically ill patients, despite the fact that CAP is one of the most frequent infections causing sepsis [7,8] and that it has a potential association with endothelial damage [9]. 

Gene expression analysis in blood is a useful tool to investigate host immune response in severe infections. Our hypothesis is that there could be transcriptomic signatures of immunological synapsis depression in non-critically ill patients with CAP with organ failure as reported in sepsis and VAP. Profiling these signatures could help to identify these patients [10]. 

To test this hypothesis we evaluated the expression levels of a number of genes involved in the immunological synapsis in patients with CAP in the presence or absence of organ failures. Additionally, two biomarkers related to endothelial dysfunction, proendothelin-1 (proET1) and proadrenomedullin (proADM), were evaluated.

## 2. Material and Methods

### 2.1. Patients and Data Collection

A prospective study was designed. Briefly, for inclusion patients had to have a new radiographic infiltrate compatible with pneumonia with at least two signs or symptoms. Nursing-home residents, human immunodeficiency virus (HIV)-positive or immunosuppressed patients, and those with limitation of therapeutic treatment, pneumonia as a terminal episode, or who had do not resuscitate orders were excluded. The ethics committee (Hospital La Fe) approved this study and patients signed informed consents. A control group was formed with 16 healthy blood donors.

We recorded data on age, gender, prior antibiotic treatment, current smoking status, alcohol abuse (>80 g/day), comorbidities (diabetes mellitus, chronic obstructive pulmonary disease (COPD), heart, liver, neurological, solid tumor, renal diseases), Pneumonia Severity Index (PSI) [11], and CURB65 (confusion; urea; respiratory rate; blood pressure; age) score. Comorbidities were defined as published in previous studies: cardiac disease (involving treatment for coronary artery disease, congestive heart failure or valvular heart disease); pulmonary disease (treatment for asthma, COPD or interstitial lung disorders); renal disease (pre-existing kidney disease with documented abnormal serum creatinine levels outside the pneumonia episode); hepatic disease (pre-existing viral or toxic liver disease); neurological disorders (presence of symptomatic acute or chronic vascular or nonvascular encephalopathy, with or without dementia); diabetes mellitus (diagnosis of glucose intolerance and treatment with oral antidiabetic drugs or insulin); and neoplastic disease (any solid tumor active at the time of presentation or requiring antineoplastic treatment within the preceding year). Charlson score [12] was also recorded in order to stratify our population.

Microbiological studies comprised blood cultures, urinary antigens for *Legionella pneumophila* and *Streptococcus pneumoniae,* sputum Gram stain, serological studies, and invasive samples and nasopharyngeal swab for viral nucleic acids if requested by the attending physician. 

### 2.2. Definitions 

Acute organ dysfunction on admission was established according to accepted criteria to define sepsis and published elsewhere [13,14,15]. Briefly, acute respiratory dysfunction was considered if oxygen saturation at admission was below 90% (or PaO2/FiO2 ratio <300); neurological dysfunction was defined as a Glasgow coma scale score below 14; acute renal dysfunction was defined as urinary debit <0.5 mL/Kg/h during 2 or more hours or creatinine >2 mg/dL or higher than 0.5 respect to basal level; tissue hypoperfusion was considered when lactate was >2mm/L or >18 mg/dL; coagulation dysfunction was defined as platelet count <100.000/mm^3^, International Normalized Ratio (INR) >1.5, or activated Partial Thromboplastin Time (aPTT) >60 s; liver dysfunction was defined as bilirubin >2 mg/dL; and cardiovascular dysfunction was considered when systolic arterial pressure was <90 mmHg or pulmonary artery pressure was <6 mmHg. 

### 2.3. Sample Collection

A sample of 2.5 mL of blood was collected in the first 24 h of CAP diagnosis using Paxgene venous blood vacuum collection tubes (Becton Dickinson, Franklin Lakes, NJ, USA). The serum and plasma samples for biomarkers determination were also obtained and frozen at −80 °C until analysis. Blood from healthy individuals was collected at the moment of donation.

### 2.4. Biomarker Determinations

C-Reactive Protein was measured using a microparticle-enhanced turbidimetric assay (CRP Gen.3, Cobas 8000, c701; Roche Diagnostics, Basel, Switzerland). ProADM and proET1 were determined by immunofluorescent assays according to manufacturer’s instructions (Thermo Scientific BRAHMS through TRACE technology in KRYPTOR systems). With the exception of proET1, all analytes were characterized by good ex vivo stability for days at room temperature (proET-1, 4 h).

### 2.5. RNA Extraction and Quality Analysis

Total RNA was extracted from blood samples using the PAXgene Blood RNA System (PreAnalytix, Hombrechtikon, Switzerland). The evaluation of concentration and quality was performed using spectrometry (Nano- Drop ND1000, NanoDrop Technologies, Wilmington, DE, USA) and the RNA Experion Bioanalyzer (BioRad, Hercules, CA, USA). 

### 2.6. Quantification of Immunological Synapse Gene Expression by Droplet Digital Polymerase Chain Reaction (ddPCR)

Genes considered in this study to interrogate the immunological synapse were previously found to be depressed in sepsis and VAP in our studies using whole genome transcriptomics analysis (PMID: 25557485, PMID: 30581823). Gene expression was quantified by ddPCR (BioRad) using predesigned TaqMan Assay Primer/Probe Sets (FAM labeled MGB probes, Thermo Fisher/Scientific-Life Technologies, Waltham, MA, USA) for HLA-DRA (major histocompatibility complex, class II, DR alpha, Hs00219575_m1); ICOS (inducible T-cell costimulator, Hs04261471_m1); CD40LG (CD40 ligand, Hs00163934_m1); CD28 (CD28 molecule, Hs01007422_m1); and CD3E (CD3e molecule, Hs01062241_m1). cDNA was generated from each sample on a Techne TC-512 thermal cycler (Bibby-Scientific, Staffordshire, OSA, UK; OSA, Washington, DC, USA) starting from 1000 ng of mRNA by using the iScript Advanced cDNA Synthesis Kit (BioRad, cat: 1725038). The obtained volume of cDNA (20 microliters) was further diluted (1/25), and 2.5 microliters (5 ng of total mRNA) were employed for quantification of target gene expression as previously described and reported [5]. Briefly, droplet digital polymerase chain reaction (ddPCR) was performed using the BioRad QX200 ddPCR system, ddPCR Supermix for Probes (no dUTP), and BioRad standard reagents for droplet generation and reading. End-point PCR with 40 cycles was performed by usingC1000Touch Thermal Cycler (BioRad) after splitting each sample into approximately 20,000 droplets. Next, the droplet reader used at least 10,000 droplets to determine the percentage of positive droplets and calculation of copy number of cDNA per nanogram of initial mRNA.

### 2.7. Outcome Measurements

The outcome measures comprised clinical stability, length of stay (LOS), and mortality (in-hospital and 30-day). Clinical stability was defined using the modified Halm criteria [16] (temperature ≤37.2 °C, heart rate ≤100 beats/min, respiratory rate ≤24 breaths/min, systolic blood pressure >90 mmHg, and oxygen saturation ≥90%) [17].

### 2.8. Statistical Study

Data analysis was performed using SPSS 15.0 software. Categorical variables were analyzed using the chi-squared test, and continuous variables with Student’s *t*-test or Mann–Whitney U test depending on whether the sample distribution was normal or not, respectively. The whole cohort was stratified in two groups: patients with at least one organ dysfunction versus those without. To evaluate genes and biomarkers the cut-off point was calculated using the area under the receiver operating curve (AUROC) to estimate the optimal operating point with highest sensitivity and specificity, as previously described [18].

Several multivariable logistic regression analyses were performed to predict CAP with organ dysfunction (the dependent variable). Each model evaluated one gene or one biomarker due to high collinearity among them. As independent variables, age, gender, Charlson score, and CURB65 were included as they were considered clinically relevant. 

The Hosmer and Lemeshow goodness-of-fit test was performed to evaluate the adequacy of the models [19].

## 3. Results

### 3.1. Clinical Characteristics 

Ninety-four patients were included in the study, 42 of whom had organ dysfunction in at least one organ. Characteristics of patients, according the presence of organ dysfunction, are depicted in Table 1. Only five patients were admitted to the intensive care unit (ICU). 

### 3.2. Gene Expression Profiles and Biomarkers

**Figure 1:**
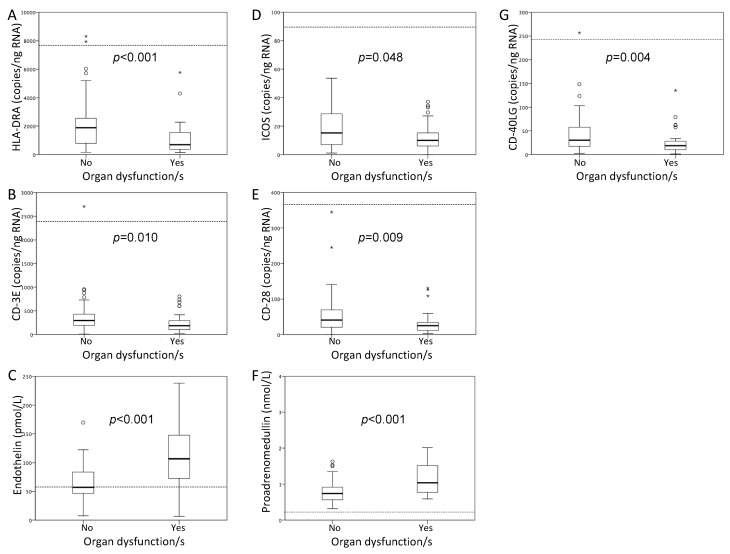
Box plots representing genes expression and biomarker levels according to the presence of organ dysfunction/s. The boxes show the interquartile range and the line inside shows the median. Dashed lines show the median values of the healthy controls. HLA-DRA: histocompatibility complex class II DR alpha; ICOS: Inducible T-cell costimulator; CD40LG: CD40 ligand (**A**) HLA-DRA. (**B**) CD3E. (**C**) Endothelin. (**D**) ICOS. (**E**) CD28. (**F**) Proadrenomedullin. (**G**) CD40LG. For comparison purposes, the Mann–Whitney U test was used.

Patients with CAP and organ dysfunction showed lower expression of the five genes evaluated and higher levels of proET1 and proADM (*p* < 0.05) (Figure 1). Expression levels of the immune synapse genes correlated directly with oxygen saturation at hospital admission and inversely with severity as assessed by the CURB65 score. The markers of endothelial dysfunction behaved just in the opposite way (Table 2). 

Gene expression and biomarkers levels depending on time to achieve clinical stability and length of hospital stay (LOS) are presented in Table 3. Patients requiring more days for achieving stability presented lower levels of CD28, CD3E, and CD40LG and higher levels of proET1 and ProADM (*p* < 0.05) (Table 3). Patients with a longer stay showed lower levels of CD28 and CD40LG and higher levels of proET1 (*p* < 0.05).

Optimal operating points (OOPs) in the AUROCs were calculated for each gene and biomarker in order to dichotomize independent variables and create categorical variables for multivariable analyses. Areas under the receiver operating curve to predict organ dysfunction were calculated for genes and biomarkers (Table 4). 

### 3.3. Multivariate Analyses 

Categorical variables were tested in a multivariable analysis (Table 5), which evidenced that low expression levels of three genes (HLA-DRA, CD40LG, and CD28) and proET1 were independent predictors of organ dysfunction at CAP diagnosis, after adjusting for age, gender, CURB65, and Charlson comorbidity index.

## 4. Discussion

This study demonstrates that non-critically ill patients with CAP and organ dysfunction present with transcriptomic signatures compatible with depression of the immunological synapse. We have found down-regulation of genes involved in central events of the adaptive immunity such as antigen presentation and T cell response. This depression is accompanied with an increase in the systemic levels of biomarkers reflecting endothelial activation and dysfunction—proET1 and proADM—and is associated with a delay in reaching clinical stability.

The concomitant presence of immunological depression and endothelial injury is a classical hallmark of sepsis. Hopp et al. have reported T-cell exhaustion in intensive care unit (ICU) patients and have proposed a stratification for severity of CAP using transcriptomic of interferon response and erythrocyte mRNA expression [20]. Interestingly, our study of non-critically ill patients hospitalized with CAP in a regular ward confirms the presence of signatures of immunological depression when organ dysfunction appears, as previously confirmed in a surgical sepsis cohort [5].

The potential detrimental impact of this depression on antigen presentation and T-cell function and its potential implications for the pathogenesis of organ dysfunction remain to be elucidated. Depressed expression of HLA-DRA in monocytes and dendritic cells has been found in patients with sepsis, probably due to an increased apoptosis of lymphoid cells or T-cell exhaustion. Zhuang et al. [9] reported that patients with mHLA-DR ≥27.2% had significantly better outcomes compared to those with levels of <27.2%. Inducible T cell co-stimulator (ICOS) is involved in directing effector T cell differentiation and has an important role in adaptive immunity [21]. T-cells express also a positive co-stimulatory molecule named CD28 that collaborates with the antigen-presenting cell providing a “second signal” that promotes T-cell activation. In contrast, a decreased expression of CD28 promotes apoptosis. CD40LG (CD154) is also a co-stimulatory molecule of activated T cells necessary for B cell activation participating in adaptive immunity and a modulator of inflammatory pathways [22].

In turn, CD3E is part of the T-cell receptor–CD3 complex, which couples antigen recognition to intracellular signal transduction pathways. Genes best performing to differentiate between presence and absence of organ dysfunction were HLA-DRA and CD40LG. 

The multivariable analysis showed that the association between gene expression levels and organ failure was robust, since it was independent of patient’s age, previous comorbidities, and severity at admission. To evaluate the impact of comorbidities we used the Charlson score that standardizes the effect of concomitant diseases and age, because it is well known that the risk of developing organ failures secondary to infection increases in elderly patients with chronic diseases. CD40LG, CD28, and HLA-DRA exhibited the highest OR to predict organ dysfunction with similar values.

Two studies have identified two gene expression signatures associated with immunoparalysis in severe CAP, similar to those found in the present work: sepsis response signature 1 (SRS1) and molecular diagnosis and risk stratification of sepsis 1 (MARS1), which predict fatal outcome [7,23].

Our study provides also new clues on the association between immunological depression and endothelial dysfunction. Recent publications reinforce the notion that sepsis alters microcirculation and disrupts the endothelial barrier [24], causing acute endothelial dysfunction and impairing hemostasis and leukocyte trafficking with recruitment of cells to infection sites. Depression of genes related to immune synapse correlated with an increase in levels of biomarkers reflecting endothelial damage, such as proET1 and proADM, which could denote a poor control of the infectious process in those patients with immuno-paralysis. These are biomarkers produced by vascular endothelial cells although it is also produced by other organs and has been associated with higher mortality in CAP. 

Our findings evidence that from the very early moments in the natural course of CAP with organ failure, patients may present signatures denoting a disturbed adaptive immunity, even in the absence of critical illness. In the next future, endotyping CAP [25] could represent a valuable tool to adequately manage patients with CAP and a new research field in infections provoking sepsis [26].

## 5. Limitations

Gene expression was profiled at diagnosis of CAP. Further evaluation at different time points following hospital admission could help to obtain a wider picture of the impact of host immune responses in CAP, along with their evolution in response to treatment, and their impact on prognosis. In fact, persistence of reduced expression of HLA-DRA is associated with a poorer outcome [27]. Due to the number of patients included, our study is not sufficiently powered to evaluate gene expression related to microbiological diagnosis.

## 6. Conclusions

Our study identifies the presence of an early depression of the genes participating in the immunological synapse, which correlates with increased endothelial injury at diagnosis of CAP requiring hospitalization, even in patients with non-critical illness. Simultaneous profiling of immunological and endothelial biomarkers could contribute to early identification of the presence of organ failure in CAP and opens a new avenue for personalizing treatment of this disease.

## Figures and Tables

**Table 1:** Demographic characteristics, comorbid conditions and prognostic scales in community-acquired pneumonia (CAP) according to organ failure.

	Organ Dysfunction		
	*n* 52 (55.3)	Yes 42 (44.7)	*p*-Value
Demographics			
Age (years)	60 (42–69)	70 (56–78)	* 0.002
Gender (male)	32 (61.5)	24 (57.1)	0.666
Pneumococcal vaccination	1 (1.9)	3 (7.1)	0.213
Influenza vaccination	14 (26.9)	16 (38.1)	0.248
Current smoker	10 (19.2)	10 (23.8)	0.081
Alcohol abuse (>80 g/day)	2 (3.8)	2 (4.8)	0.179
Prior antibiotic treatment (last month)	22 (42.3)	14 (33.3)	0.374
Comorbid condition			
Diabetes mellitus	3 (5.8)	9 (21.4)	* 0.024
Chronic liver disease	0 (0)	1 (2.4)	0.263
Heart disease	13 (25)	15 (35.7)	0.259
Renal disease	2 (3.8)	5 (11.9)	0.139
Neurological disease	2 (3.8)	5 (11.9)	0.139
COPD	3 (5.8)	15 (35.7)	* <0.001
Charlson comorbidity index	0 (0–1)	1 (1–2)	* <0.001
Prognostic scales			
PSI (IV–V)	6 (11.5)	18 (42.9)	* 0.001
CURB65	1 (0–1)	2 (1–2)	* <0.001
Analytical parameters on admission			
C-reactive protein (mg/dL)	15.07 (6.32–25.32)	23.64 (9.65–33.44)	* 0.046
Leukocytes (cell/mm^3^)	12955 (8470–16800)	15115 (10330–19710)	0.090
Neutrophils (%)	81.3 (75.7–84.8)	87 (81.6–90.3)	* 0.001
Neutrophils (cell/mm^3^)	10692 (6481–14203)	13561 (7735–17597)	* 0.043
Lymphocytes (%)	8.8 (6.6–12.8)	6.8 (4.2–8.9)	* 0.005
Lymphocytes (cell/mm^3^)	1061 (795–1518)	854 (589–1292)	0.057
Outcomes			
Length of stay (days)	6 (4–8)	7 (5–10)	0.018
Clinical stability achieved	52 (100)	38 (90.5)	* 0.023
Time to clinical stability (days)	2 (1–3)	5 (3–7)	* <0.001
Treatment failure	2 (3.8)	6 (14.3)	0.071
30-days mortality	0 (0)	2 (4.8)	0.112

Data presented as *n* (%) or median (interquartile range); COPD: chronic obstructive pulmonary disease; CURB65: confusion, urea, respiratory rate, blood pressure, 65 years; PSI: pneumonia severity index. * *p* < 0.05.

**Table 2:** Spearman correlations between genes and biomarkers with CURB65 and oxygen saturation.

	CD40LG	HLA-DRA	CD28	CD3E	ICOS	ProET1	ProADM
**CURB65**	−0.365 **	−0.321 **	−0.294 **	−0.277 **	−0.274 **	0.412 **	0.608 **
**O2 saturation**	0.350 **	0.327 **	0.305 **	0.218 *	0.266 *	−0.392 **	−0.403 **
**ProET1**	−0.466 **	−0.485 **	−0.454 **	−0.477 **	−0.446 **		0.718 **
**ProADM**	−0.322 **	−0.302 **	−0.302 **	−0.305 **	−0.250 *	0.718 **	

CURB65: confusion, urea, respiratory rate, blood pressure, 65 years; O2: oxygen; ProET1: proendothelin-1; ProADM: proadrenomedullin; HLA-DRA: histocompatibility complex class II DR alpha; ICOS: Inducible T-cell costimulator; CD40LG: CD40 ligand. * *p* < 0.05. ** *p* < 0.01.

**Table 3:** Gene expression and biomarkers according to time to clinical stability and length of stay.

	Time to Clinical Stability			Length of Stay		
	≤3 Days	>3 Days	*p*-Value	≤6 Days	>6 Days	*p*-Value
HLA-DRA (copies/ng RNA)	1448 (560–2412)	924 (448–1944)	0.142	1376 (560–2360)	920 (448–1952)	0.188
ICOS (copies/ng RNA)	15 (7–27)	11 (5–19)	0.087	12 (8–25)	11 (5–19)	0.180
CD40LG (copies/ng RNA)	30 (19–59)	17 (8–28)	* 0.001	29 (16–57)	19 (8–28)	* 0.008
CD3E (copies/ng RNA)	270 (185–432)	202 (100–311)	* 0.040	270 (185–373)	199 (99–368)	0.105
CD28 (copies/ng RNA)	34 (21–65)	24 (12–37)	* 0.011	31 (21–58)	24 (17–43)	* 0.048
ProET1 (pmol/L)	69 (47–101)	89 (62–129)	* 0.027	72 (48–94)	85 (55–137)	* 0.048
ProADM (nmol/L)	0.76 (0.61–0.95)	1 (0.75–1.45)	* 0.018	0.77 (0.61–1.08)	0.9 (0.7–1.23)	0.163

Data presented as median (interquartile range). For the analysis, only surviving patients at 30 days were considered. ProET1: proendothelin-1; ProADM: proadrenomedullin; HLA-DRA: histocompatibility complex class II DR alpha; ICOS: Inducible T-cell costimulator; CD40LG: CD40 ligand * *p* < 0.05.

**Table 4:** Areas under the receiver operating curve for assessing the performance of gene expression levels and biomarkers and their optimal operating points to rule out organ dysfunction.

	AUROC	*p*-Value	OOP	Sensitivity	Specificity
HLA-DRA	* 0.719 (0.616-0.822)	* <0.001	1204 copies/ng RNA	0.635	0.738
ICOS	* 0.619 (0.505-0.733)	* 0.048	13 copies/ng RNA	0.577	0.714
CD40LG	* 0.671 (0.562-0.780)	* 0.005	29 copies/ng RNA	0.538	0.833
CD3E	* 0.654 (0.542-0.766)	* 0.010	234 copies/ng RNA	0.635	0.667
CD28	* 0.657 (0.546-0.768)	* 0.009	38 copies/ng RNA	0.538	0.833
ProET1	0.775 (0.663-0.886)	* <0.001	87 pmol/L	0.676	0.795
ProADM	0.756 (0.646-0.865)	* <0.001	0.9 nmol/L	0.618	0.744

AUROC: area under the receiver operating curve; OOP: optimal operating point; ProET1: proendothelin-1; ProADM: proadrenomedullin; HLA-DRA: histocompatibility complex class II DR alpha; ICOS: Inducible T-cell costimulator; CD40LG: CD40 ligand * *p* < 0.05.

**Table 5:** Multivariable analysis evaluating the association between gene expression levels below each respective OOP and risk of presenting with organ failure/s adjusted for age, sex, Charlson comorbidity index, and CURB65.

			Age		Gender		Charlson Comorbidity Index		CURB65	
	OR (95% CI)	*p*	OR (95% CI)	*p*	OR (95% CI)	*p*	OR (95% CI)	*p*	OR (95% CI)	*p*
Genes										
HLA-DRA (<OOP)	2.94 (1.07–8.09)	0.037	1.01 (0.97–1.05)	0.728	1.35 (0.48–3.79)	0.565	1.41 (0.86–2.32)	0.171	1.82 (0.98–3.39)	0.058
ICOS (<OOP)	2.06 (0.76–5.58)	0.154	1.01 (0.97–1.05)	0.737	1.60 (0.59–4.34)	0.355	1.48 (0.90–2.41)	0.121	1.92 (1.04–3.54)	0.038
CD40LG (<OOP)	3.90 (1.34–11.37)	0.013	1.00 (0.96–1.04)	0.924	1.54 (0.56–4.22)	0.398	1.48 (0.90–2.43)	0.120	1.91 (1.02–3.60)	0.044
CD3E (<OOP)	2.42 (0.92–6.34)	0.072	1.01 (0.97–1.05)	0.766	1.61 (0.60–4.31)	0.347	1.48 (0.91–2.41)	0.119	1.96 (1.05–3.67)	0.035
CD28 (<OOP)	3.48 (1.17–10.34)	0.025	1.01 (0.97–1.05)	0.777	1.52 (0.56–4.15)	0.416	1.38 (0.84–2.27)	0.210	1.86 (1.00–3.47)	0.050
Biomarkers										
ProET1 (>OOP)	6.50 (1.93–21.88)	0.002	0.99 (0.95–1.04)	0.750	0.80 (0.26–2.42)	0.688	1.21 (0.71–2.05)	0.490	1.45 (0.71–2.96)	0.306
ProADM (>OOP)	2.42 (0.78–7.53)	0.127	1.02 (0.97–1.06)	0.566	0.84 (0.29–2.42)	0.280	1.34 (0.79–2.26)	0.746	1.23 (0.61–2.46)	0.478

OR: odds ratio; CI: confidence interval; CURB65: confusion, urea, respiratory rate, blood pressure, 65 years; OOP: optimal operating point; ProET1: proendothelin-1; ProADM: proadrenomedullin; HLA-DRA: histocompatibility complex class II DR alpha; ICOS: Inducible T-cell costimulator; CD40LG: CD40 ligand.
